# A Pilot Study to Improve Cognitive Performance and Pupil Responses in Mild Cognitive Impaired Patients Using Gaze-Controlled Gaming

**DOI:** 10.3390/vision8020025

**Published:** 2024-04-24

**Authors:** Maria Solé Puig, Patricia Bustos Valenzuela, August Romeo, Hans Supèr

**Affiliations:** 1 Unitat d’Avaluació de la Cognició, l’Atenció i l’Aprenentatge (ACAP), 08035 Barcelona, Spain; mariasoleacap@gmail.com; 2 Vision and Control of Action Group, Department of Cognition, Development and Educational Psychology, University of Barcelona, 08035 Barcelona, Spain; pbustova19@alumnes.ub.edu (P.B.V.);; 3 Institute of Neurosciences, University of Barcelona (UBNeuro), 08035 Barcelona, Spain; 4 Research Institute Sant Joan de Déu (IRSJD), 08950 Barcelona, Spain; 5 Braingaze SL, 08302 Mataró, Spain; 6 Catalan Institution for Research and Advanced Studies (ICREA), 08010 Barcelona, Spain

**Keywords:** attention, digital treatment, MCI, visual oddball, pupil

## Abstract

Mild cognitive impairment (MCI) may progress to severe forms of dementia, so therapy is needed to maintain cognitive abilities. The neural circuitry for oculomotor control is closely linked to that which controls cognitive behavior. In this study, we tested whether training the oculomotor system with gaze-controlled video games could improve cognitive behavior in MCI patients. Patients played a simple game for 2–3 weeks while a control group played the same game using a mouse. Cognitive improvement was assessed using the MoCA screening test and CANTAB. We also measured eye pupil and vergence responses in an oddball paradigm. The results showed an increased score on the MoCA test specifically for the visuospatial domain and on the Rapid Visual Information Processing test of the CANTAB battery. Pupil responses also increased to target stimuli. Patients in the control group did not show significant improvements. This pilot study provides evidence for the potential cognitive benefits of gaze-controlled gaming in MCI patients.

## 1. Introduction

Mild cognitive impairment (MCI) is a condition that affects cognitive function, including attention, memory, and executive function. While MCI does not typically interfere significantly with daily activities, it may progress to more severe forms of cognitive decline, such as dementia [1,2,3,4,5,6]. As such, there is a need for therapy or treatment for patients with MCI to maintain their mental abilities and potentially delay the onset of more severe cognitive impairment.

Cognitive improvement may involve various approaches, such as cognitive training, physical exercise, and diet modifications, and there is growing interest in the use of computerized tasks and video games as a potential intervention to improve cognitive functions. Research has suggested that video gaming and computerized task can improve attention, working memory, executive function, and visuospatial abilities [7,8,9,10,11,12,13,14]. Enhancements have been observed not only in healthy individuals, but also in patient populations with attention problems [15], including MCI patients [16,17,18]. However, meta-analysis studies revealed small to moderately positive treatment effects in MCI patients [13,19,20,21,22], and some studies found no positive effects of computerized training tasks in healthy participants or in MCI patients [19,23,24]. Thus, although video games are a promising technology, they need to be further developed to become a tool for intervention.

Patients with cognitive disorders typically demonstrate altered pupil responses [25,26] and oculomotor deficits [26,27,28,29,30,31]. Ample evidence shows that modulation in pupil size manifest cognitive processing and likely reflects the functioning of the locus coeruleus [32,33,34], which is a key structure in attentional processing. In addition to pupil responses, eye movements play a role in perception and attention. [35,36,37,38,39,40]. It is thus plausible that the oculomotor system can be used as a means of cognitive intervention. Indeed, recent evidence shows that training the oculomotor system with gaze-controlled video games can be effective in improving attention in ADHD patients [41]. The goal of this study is to determine whether gaze-controlled games can improve cognitive behavior in patients with MCI.

## 2. Materials and Methods

### 2.1. Participants

A total of 50 patients with MCI were recruited by the coordinators from three different private day care centers in Barcelona, Spain. As our primary aim was to see whether a gaze-controlled game improves attention processing, we first recruited patients with MCI to assess cognitive improvement. The recruitment of the control participants was performed after testing the patients. Unfortunately, as we needed to finish the study, the control group was not as large as the experimental group. Thirteen patients withdrew from the study before completion because of personal reasons, and following the protocol their data were excluded from the analysis. Therefore, no intent-to-treat analysis could be performed. Of the remaining 37 patients, 29 (19 female) participated in the experimental group and 8 (5 female) were in the control group. Participants were 63–86 years old (mean ± SD: 77.91 ± 6.85). All participants had a history of cognitive decline, confirmed by their Montreal Cognitive Assessment (MoCA) scores (see Results). The exclusion criteria were as follows: (1) history of neurological disease with clinically relevant impact on cognition (e.g., cerebrovascular disease); (2) severe psychiatric disorder; (3) presence of relevant visual problems; and (4) problems for understanding spoken or written Spanish language.

### 2.2. Ethics Statement

Participants and their relatives received detailed instructions for the experiments. Prior to enrollment, patients or their relatives signed a written informed consent for their participation, in accordance with the tenets of the Declaration of Helsinki. The ethics committees of the University of Barcelona approved the study.

### 2.3. Video Game

**Figure 1:**
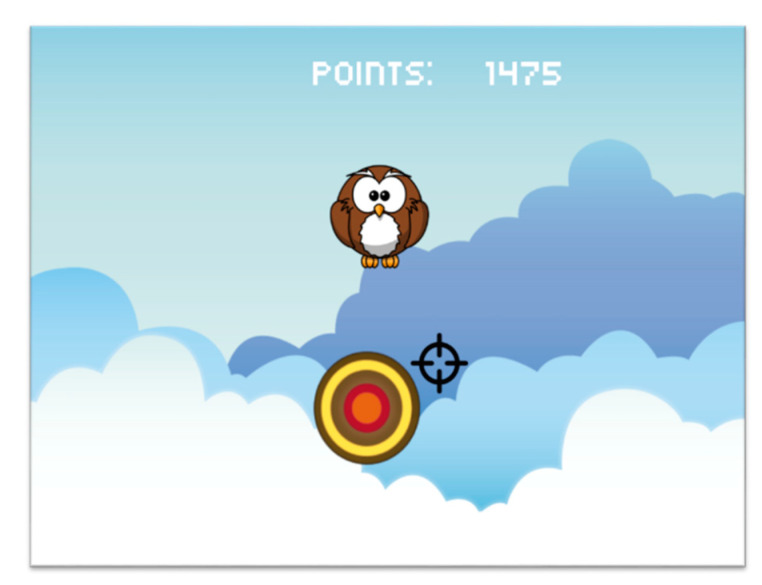
Illustration of the video game. Note that in the actual game, only 1 target (dartboard) or distractor (owl) was presented. The pointer indicated the position of the eye gaze or mouse cursor in real time.

The video game (Figure 1) consisted of a stationary or moving (left to right or vice versa) target (dartboard-like picture) and a distractor (picture of an owl). Participants had to look at or follow the moving target with their eyes for 1 s and avoid looking at the (moving) distractor. If successful, the target would “explode” and the participant received points, or the owl would disappear. If unsuccessful, the target would disappear or the owl would “explode”, and the participant would lose points. Only one target or distractor was presented at a time. Eye position was recorded with a remote eye tracker (5L, Tobii, Danderyd, Sweden) and fed back to the presentation software to control the game. The actual eye position was indicated by a pointer on the screen. In the control group, participants played the same game but used a mouse cursor as a controller instead of eye gaze. Participants played 3 sessions per week for approximately 15 min each over a period of one month.

### 2.4. Neuropsychological Pre and Post Assessment Instruments

Global cognitive performance of participants was assessed using the Montreal Cognitive Assessment (MoCA) and the Cambridge Neuropsychological Test Automated Battery (CANTAB^®^; Cambridge Cognition Ltd., Cambridge, UK). In addition, a visual oddball paradigm was applied to assess pupil and vergence responses [26].

#### 2.4.1. MoCA

MoCA is a widely used cognitive screening tool that assesses various aspects of cognitive function, including attention, memory, language, visuospatial abilities, and executive function [15]. It was designed to detect MCI and early dementia in adults. The maximum score on the test was 30, with a score of 26 or above considered normal.

#### 2.4.2. CANTAB

CANTAB is a computerized cognitive assessment battery. The CANTAB battery consists of a series of tests that assess various cognitive domains, including attention, memory, executive function, and visuospatial abilities. The battery has been extensively validated and is widely used in both research and clinical settings to assess cognitive function in a range of populations including MCI [42].

For this study we used the following tests:

- Rapid visual information processing (RVP) is a measure of sustained attention.
- Paired associates learning (PAL) assesses visual memory and new learning.
- The motor screening task (MST) provides a general assessment of sensorimotor deficits.
- Pattern recognition memory (PRM) is a test of visual pattern recognition memory in a 2-choice forced discrimination paradigm.
- Reaction time (RT) provides assessments of motor and mental response speeds, as well as measures of movement time, reaction time, response accuracy, and impulsivity.
- Spatial working memory (SWM) requires the retention and manipulation of visuospatial information.
- Delayed matching to sample (DMS) assesses both simultaneous visual matching ability and short-term visual recognition memory.

#### 2.4.3. Visual Oddball Paradigm

To assess possible changes in pupil and eye vergence responses, patients performed a visual oddball task before and after the gaming sessions [26]. The task was a sequence of 100 trials. Each trial started with a grey screen (Mask) for 2000 ms, followed by the stimulus screen for 2000 ms. The central stimulus consisted of a series of randomly selected letters forming a string of 11 characters in upper or lower case. Letter strings did not represent acronyms or meaningful words. In the distractor condition, the color of all characters of the string was blue (80% of trials), whereas in the target condition, the color of the characters was red (20% of trials). Target and distractor stimuli were randomly presented. Participants were instructed to press a response button when the characters of a string appeared in red. The total duration of the task was about 6 min.

The BGaze (Braingaze SL, Mataró, Spain) system was used to present the visual oddball task. Eye position data were recorded with an X2-30 (30 Hz) remote eye tracker (Tobii Technology AB, Danderyd, Sweden) mounted below the screen. The screen resolution was 1024 × 768 pixels. Patients were seated 50–60 cm from the stimulus screen. Patients could wear corrective lenses. Before starting the recording, the eye tracker was calibrated (5 points, binocular) for each participant. No chinrest was used during the task.

The eye data obtained during the visual oddball task were used to calculate pupil and vergence responses. In order to calculate vergence changes, we transformed the coordinates of the left and right eye, supplied by the eye tracker, into angular magnitudes (degrees). The subtraction of initial values from every response, which was applied both to vergence and to pupil data, served the purpose of obtaining relative changes. Only correct trials were analyzed.

### 2.5. Statistical Analysis

One-tailed *t*-tests were used to evaluate improvements in MoCA scores. Raw scores of CANTAB test measures were selected for statistical evaluation. For comparisons between baseline and post-game CANTAB scores, the one-tailed Wilcoxon signed rank and Kruskal–Wallis ANOVA tests were used. In addition, we used analyses of variance (ANOVA) with repeated measures. For the analyses of pupil and vergence responses, two sided paired *t*-tests (Welch) were applied. The significance level was set at *p* < 0.05. MATLAB (MathWorks) was used for data and statistical analysis.

## 3. Results

### 3.1. Video Gaming

On average, 11.7 ± 2.20 (mean ± SD; min: 9; max: 16) sessions were completed. The mean session duration was 16.12 ± 3.22 min (mean ± SD). The mean proportion of correctly identified targets was 55.25%. Participants did not improve over the course of therapy, as there was no significant (*p* > 0.05) correlation (Spearman rank) between the number of sessions played and the number of correct responses (R^2^ = 0.21).

### 3.2. MoCA

**Figure 2:**
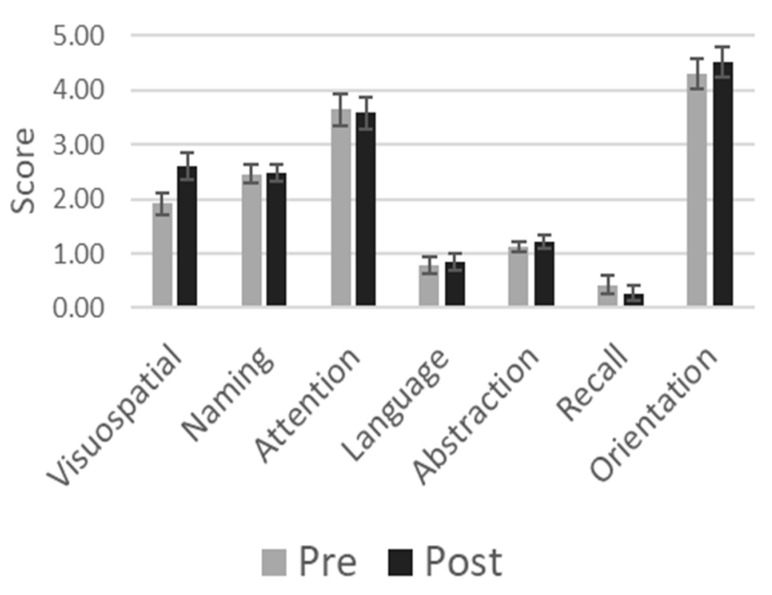
MoCA scores obtained before (pre) and after (post) the gaming sessions.

**Figure 3:**
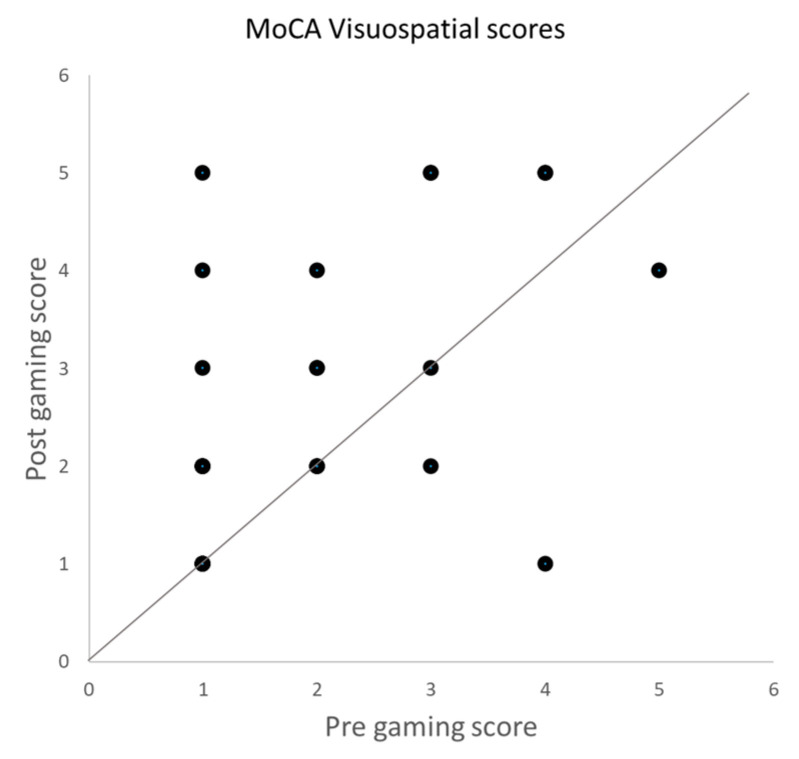
Scores of the MoCA in the visuospatial obtained before and after the gaming sessions. Each dot represents the score of one participant.

The total MoCA score improved from 14.64 ± 4.28 (mean ± SD) at baseline to 15.52 ± 4.76 after gaming (*p* < 0.03). When comparing the scores for the different items, we observed that the improvement was significant in the visuospatial domain (*p* < 0.002) and remained significant after Bonferroni correction for multiple comparison. The results of the MoCA assessment per cognitive function tested are shown in Figure 2. The effect sizes are small for total MoCA scores (Hedges’ g factor: 0.20) and moderate for the visuospatial domain (Hedges’ g factor: 0.52). Of the participants, 55% improved and 10% worsened in this domain (Figure 3). None of the other domains showed significant improvement.

There was no significant correlation (Spearman rank) between the number of video game sessions played and improvement in either the overall MoCA score (R^2^ = 0.067) or the visuospatial domain score of the MoCA (R^2^ = 0.155). Neither there was a significant correlation (R^2^ = 0.001) between the improvement in the overall MoCA score and the pre–post training difference in detection performance of the video game. We repeated the calculation using the MoCA scores in the visual domain; neither found a significant correlation (R^2^ = 0.012).

### 3.3. CANTAB

**Figure 4:**
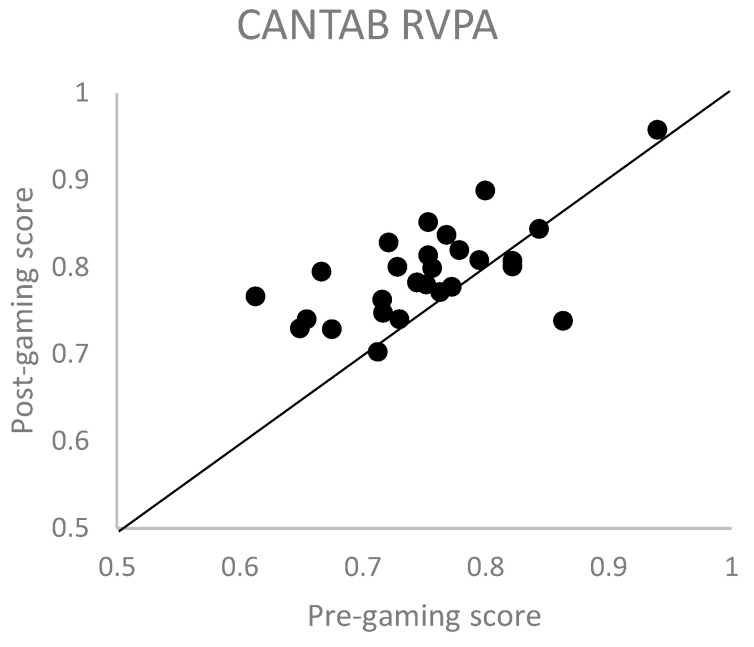
Rapid visual information processing (RVPA) scores of the CANTAB obtained before and after the gaming sessions. Each dot represents the score of one participant.

Of the various CANTAB tests used, only rapid visual information processing (RVP) showed significant improvements after the end of the game session (Table 1). The most significant improvement was observed in participant’s sensitivity (RVPA), which is a measure of how well the participant is able to detect target sequences (Figure 4). The effect size for RVPA was 0.67 (Hedges’ g-factor). Except for a few scores, all other tests (paired associates learning, motor screening task, pattern recognition memory, reaction time, spatial working memory, and delayed matching to sample) showed no statistically significant improvement after the gaming sessions (Tables S1–S5 in Supplementary Materials).

### 3.4. Control Group

To assess whether the improvements observed on the MoCA test and the RVP task of the CANTAB battery were specific to the gaze-controlled game, we tested a small group of MCI patients who played the same game but controlled it with a mouse instead of their eyes.

**Figure 5:**
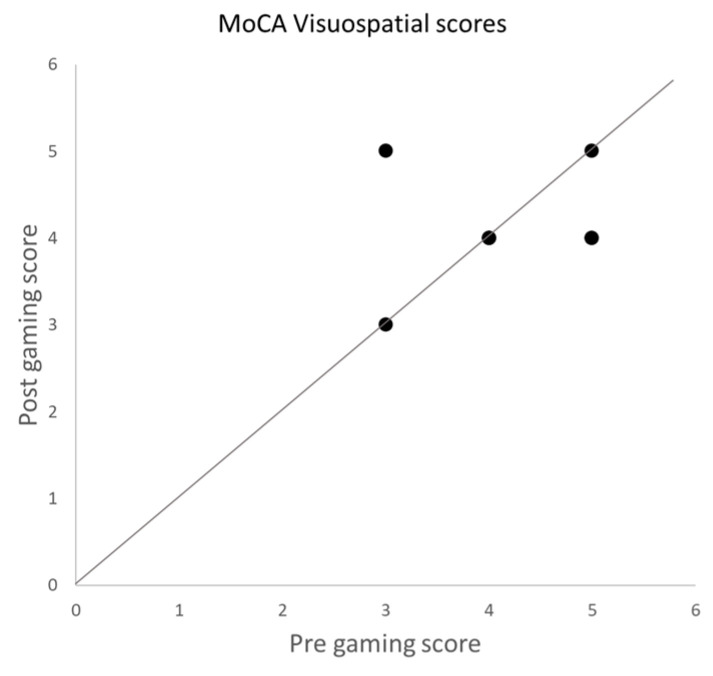
Scores of the MoCA in the visuospatial domain obtained before and after the gaming sessions. Each dot represents the score of one participant.

Overall MoCA scores did not improve significantly between baseline (mean ± SD: 22.50 ± 6.82) and post-game (23.00 ± 6.93; *p* = 0.37). No significant changes were observed in any of the individual domains of the MoCA, including the visuospatial domain (mean ± SD: baseline: 4.13 ± 0.83; post-gaming: 4.13 ± 0.64; *p* = 0.5). Of the participants, 12.5% improved and 25% worsened in this domain (Figure 5). Note that in the control group, the overall MoCA scores were higher than in the experimental group. A Wilcoxon rank sum test showed significant differences in the MoCA scores before (*p* < 0.0002) and after (*p* = 0.0003) gaming sessions between the experimental and control group. The RVP task of the CANTAB battery also showed no significant differences in any of the measures. For example, sensitivity (RVPA) at baseline was 0.84 ± 0.07 and 0.81 ± 0.10 (mean ± SD) after the game (*p* = 0.8309). On the other administered CANTAB tasks, no significant improvements were observed.

Since the pre-test scores were higher in the control group than in the experimental group, a ceiling effect may have influenced the results. Therefore, we divided the MoCA scores in the visuospatial domain of the experimental group into ‘low’, ‘mid’, and ‘high’, depending on their score. Then, ANOVA was applied to the factor ‘group’ (‘low’, ‘mid’, ‘high’, and ‘control’), in addition to the factor ‘session’ (‘pre’, ‘post’). The marginal means for the ‘group’ factor were 1.44 ± 0.86, 1.67 ± 1.03, 3.67 ± 1.08, and 4.12 ± 0.72 for ‘low’, ‘mid’, ‘high’, and ‘control’, respectively. Significant differences were present (F(3,62) = 38.7, *p* = 3,2 10-14), and the Tukey–Kramer procedure indicated that ‘low’–‘high’, ‘mid’–‘high’, ‘low’–‘control’, and ‘mid’–‘control’ differences were significant. The session factor involved significant differences as well (F(1,62) = 5.23, *p* = 0.026). After restricting session comparisons to each of the three patient groups, we observed that the significant difference between ‘pre’ and ‘post’ sessions came from the ‘low’ patient group (‘pre’: 1.00 ± 0.00, ‘post’: 1.89 ± 1.05, F(1,16) = 6.4, *p* = 0.022) and not from ‘mid’ (‘pre’: 1.33 ± 0.50, ‘post’: 2.00 ± 1.32, F(1,16) = 2.0, *p* = 0.18) or ‘high’ (‘pre’: 3.44 ± 0.88, ‘post’: 3.89 ± 1.27, F(1,16) = 0.74, *p* = 0.40) patient groups.

### 3.5. Visual Oddball Paradigms and Pupil and Vergence Responses

**Figure 6:**
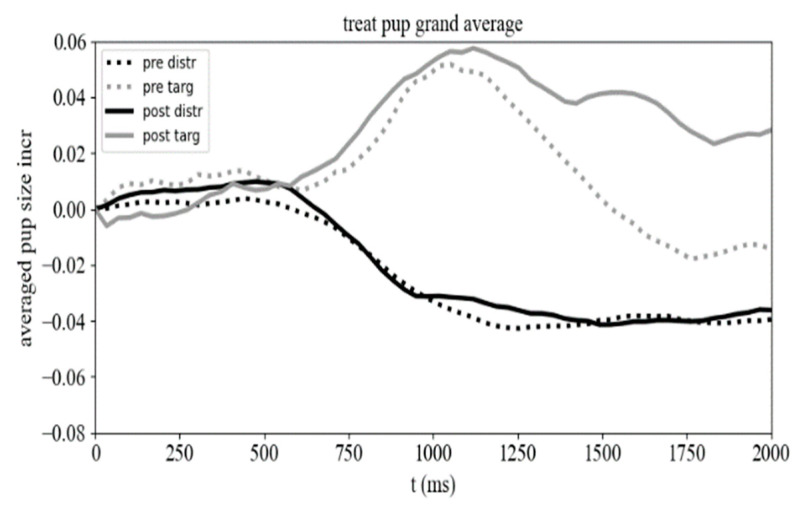
Pupil responses before (pre) and after (post) the gaming sessions to targets (targ) and distractors (distr).

We first looked at the behavioral performance of both groups in the oddball task. The overall performance was 96% and 94% correct in the control and patient group, respectively. No significant differences were observed between groups and pre–post sessions. We next evaluated whether pupil size and vergence responses had changed after the gaming sessions. We observed modulatory pupil responses where responses were stronger to targets than to distractors (Figure 6). After gaming, there was a significant increase in the late period (average window of 1500–2000 ms) of the pupil responses to targets (mean ± SD: pre: −0.012 ± 0.24; post: 0.032 ± 0.17; *t* = −2.41, *p* = 0.016). Responses to distractor stimuli did not change after gaming (mean ± SD: pre: −0.034 ± 0.168; post: −0.039 ± 0.155; *t* = −0.064, *p* = 0.94).

Vergence responses to target stimuli were not significantly (*t* = −0.51, *p*= 0.61) different after gaming (mean ± SD: 0.186 ± 2.34) compared to baseline responses (mean ± SD: 0.062 ± 3.33). Neither distractor responses showed significant changes (mean ± SD: pre: −0.034 ± 2.77; post: 0.15 ± 2.35; *t* = −1.9, *p* = 0.057). The control group showed comparable pupil responses to distractors (mean ± SD: pre: −0.050 ± 0.16; post: −0.069 ± 0.16; *t* = 0.838, *p* = 0.109) and targets (mean ± SD: pre: 0.032 ± 0.18; post: 0.014 ± 0.18; *t* = 0.87, *p* = 0.39) after gaming. Moreover, vergence responses were similar before and after the gaming sessions (target, mean ± SD: pre: 0.16 ± 2.6; post: 0.075 ± 1.74; *t* = 0.35, *p* = 0.73; distractor: mean ± SD: pre: 0.12 ± 2.32; post: 0.18 ± 1.89; *t* = −0.50, *p* = 0.61).

ANOVA showed that for pupil responses and distractors, the difference caused by the interaction between group (experimental, control) and session (pre-, post-gaming) was not significant (*p* = 0.102). However, in the case of target stimuli, it was significant (*p* = 0.049). The ‘group’–‘session’ interaction for vergence responses did not produce any significant difference (distractors: *p* = 0.496, targets: *p* = 0.624). We performed a Kruskal–Wallis ANOVA to compare the effect of gaming on MoCA performance in gaze and mouse controlled condition, and found a significant effect of gaming (F(3,82) = 26,51, *p* = 7 × 10^−6^).

## 4. Discussion

In the current study, we tested a gaze-controlled video game as a potential intervention tool to improve cognitive function in MCI patients. The gaze-directed game required participants to repeatedly maintain focus on a target for short periods of time, thus training sustained attention. The results show an increased score on the MoCA test specifically for the visuospatial domain after playing the game. This may indicate that the improvement with the gaze-controlled game is task-specific, possibly due to the visual and spatial demands of the video game. Of the CANTAB battery, the rapid visual information processing test was the only test to show improvement. Again, the improvement may be task-specific. The rapid visual information processing test primarily assesses the ability to focus and maintain attention on a task over time. The other CANTAB test we used primarily tested short-term memory, which was not trained by the game. The results of the current report support our previous findings of cognitive improvement with gaze-controlled games [41], and are consistent with previous studies [16,17,18] showing that video games can have a positive effect on cognitive function.

Practice effects cannot be ruled out, as the MoCA test may be susceptible to practice effects in healthy older adults ([43], but see [44]) and MCI patients [45]. The CANTAB battery tests also show practice effects in patients with cognitive decline. In particular, the paired associates learning, spatial working memory, and motor screening tests are associated with large practice effects [46,47]. This is in contrast to our observations, which show no practice effects on these tasks. The rapid visual information processing and reaction time tests show no or weak practice effects in MCI patients [46].

Thus, our findings suggest that the improvements observed in the current study are a true effect of playing a gaze-controlled game. This assumption is supported by the outcomes of the control group, in which participants showed no practice effects on the rapid visual information processing tests, nor on any of the tests administered. However, the size of the control group was small, and their pre-test scores were relatively high compared to the experimental group, which may have introduced a ceiling effect, making it more challenging to observe improvements.

### 4.1. Pupil and Vergence Responses

Both pupil and vergence responses are manifestations of cognitive processing. Pupil dilation reflects the allocation of top-down attentional resources [48] and has been linked to various cognitive processes, including perception [49], cognitive effort [50], memory [51,52], prediction error [53], and decision-making [33,54,55]. Similarly, vergence responses are associated with attention and memory [40,56,57], and may trigger pupil responses [49]. Our results on pupil responses are consistent with previous findings that show greater pupil dilation for target stimuli compared to distractor stimuli [26,33,58]. This suggests that the larger pupil responses to targets in the experimental group may indicate a higher level of attention or extra cognitive effort to achieve performance [59,60].

### 4.2. Neurobiological Relevance

One way in which training with gaze control tasks may impact cognitive functioning is through its impact on the locus coeruleus. The locus coeruleus is a small nucleus located in the pons of the brainstem and forms part of the reticular activating system. It is the main site for the synthesis of norepinephrine, and via its widespread connections throughout the brain, the locus coeruleus influences many brain functions. As the main source of norepinephrine to the pre-frontal cortex, the locus coeruleus also has an impact on cognitive processes that sub-serve executive functions. Within the brainstem, the locus coeruleus connects to the Edinger–Westphal nucleus. In the Edinger–Westphal nucleus, pre-ganglionic neurons project to postganglionic neurons in the ciliary ganglion, which, in turn, innervate smooth muscle fibers in the sphincter muscle of the iris to control pupil size. The Edinger–Westphal preganglionic neurons receive input from the olivary pretectal area conveying pupillary light reflex input, and from neurons of the central mesencephalic reticular formation that have a role in vergence eye movements [61]). Additionally, the superior colliculus is reciprocally connected to the Edinger–Westphal [62,63]). The Edinger–Westphal cells further receive monosynaptic excitatory input from ventral hippocampal cells, which are critical for attention [64]. These hippocampal cells innervate the medial prefrontal cortex. This area is known to control attention processing [64] and eye vergence (e.g., [65]). Input to the locus coeruleus originates from structures involved in horizontal eye movement control, such as the medial prefrontal cortex [65,66] and the nucleus prepositus [67]. Consequently, vergence eye movements and pupil responses may influence the functioning of the locus coeruleus. Future work utilizing in vivo imaging of the locus coeruleus may provide evidence for this idea [68]. Training of the oculomotor system, in general, may then positively affect the attention system.

### 4.3. Shortcomings

The study included a small number of participants in the control group. Thus, although video gaming provides positive outcomes on attention, more research is needed to conclude whether this is due to video gaming, oculomotor training, or practice effects. Further studies are needed with a higher number of participants with different types of dementia who score low on MoCA to provide clear evidence.

### 4.4. Conclusions

In conclusion, the present results provide evidence for the potential cognitive benefits of gaze-controlled gaming in MCI patients and suggest that further research in this area is warranted.

## Figures and Tables

**Table 1:** Measures obtained from the rapid visual information processing (RVP) test, presented as the mean ± SD of the raw score. After Bonferroni correction for multiple comparison, the results of the RVPA remained significant.

Type	Pre-Treatment Mean (SD)	Post-Treatment Mean (SD)	Participants N	Test Statistics		
				Ties	Z	*p*
RVPML	887.00(340.36)	787.89(205.26)	29	0	1.6866	0.0458
RVPLSD	409.24(138.68)	337.10(145.22)	29	0	2.1842	0.0145
RVPA	0.75(0.07)	0.79(0.05)	27	2	−3.4476	<0.001
RVPTH	16.59(10.29)	22.89(15.58)	27	2	−2.1403	0.0162
RVPTFA	35.81(36.73)	65.30(99.65)	25	3	−0.4858	0.3136
RVPPH	0.31(0.19)	0.42(0.29)	27	2	−1.9646	0.0247
RVPTM	34.83(13.84)	30.59(15.63)	27	2	1.5505	0.0605

## Data Availability

The original contributions presented in the study are included in the article/Supplementary Material, further inquiries can be directed to the corresponding author/s.

## Supplementary Materials

The following supporting information can be downloaded at: https://www.mdpi.com/article/10.3390/vision8020025/s1, Table S1: Measures obtained from the Paired Associates Learning (PAL) test, presented as Mean ± SD of the Raw Score; Table S2: Measures obtained from the Motor Screening Task (MOT) and Pattern Recognition Memory (PRM) test, presented as Mean ± SD of the Raw Score; Table S3: Measures obtained from the Reaction Time (RTI) test, presented as Mean ± SD of the Raw Score; Table S4: Measures obtained from the Spatial Working Memory (SWM) test, presented as Mean ± SD of the Raw Score; Table S5: Measures obtained from the Delayed Matching to Sample (DMS) test, presented as Mean ± SD of the Raw Score.
