# Supplementary material for: A Pilot Study to Improve Cognitive Performance and Pupil Responses in Mild Cognitive Impaired Patients Using Gaze-Controlled Gaming
Source: Vision (Basel). 2024 Apr 24;8(2):25. doi: 10.3390/vision8020025 (PMC11130921; doi:10.3390/vision8020025)
Supplement: Supplementary file 1 [file vision-08-00025-s001.zip › vision-2752027-supplementary.pdf]

Supplementary Materials

Table S1. Measures obtained from the Paired Associates Learning (PAL) test, presented as Mean ± SD of the Raw Score.

Type	Pre-treatment	Post-treatment	Participants	Test statistics		
	Mean(SD)	*Mean(SD)*	*N*	*Ties*	*Z*	*P*
PALTEA28	53.45(10.29)	56.83±10.52	29	0	−1.5468	0.0610
PALTA28	7.21(2.55)	6.83(1.64)	26	3	0.6740	0.2501
PALFAMS28	3.69(0.47)	2.83(0.56)	21	8	1.4664	0.0713
PALMETS28	2.10(2.49)	2.35(1.83)	19	10	0.0716	0.4714
PALNPR28	5.24(1.88)	4.55(1.40)	16	13	1.6846	0.0460
PALTEA2	1.52(2.27)	2.21(2.33)	20	9	−0.9600	0.1685
PALTEA4	7.48(4.90)	9.14(4.42)	25	4	−1.8260	0.0339
PALTEA6	17.14(4.42	17.72(4.50)	16	13	−0.8548	0.1963
PALTEA8	27.31(2.46)	27.76(1.29)	4	25	0.2981	0.3125
PALTE28	17.55(8.44)	15.34(5.95)	27	2	1.1299	0.1292
PALTE2	1.52(2.27)	2.21(2.33)	21	8	0.9600	0.1685
PALTE4	7.03(4.97)	7.79(5.00)	26	3	−0.8158	0.2073
PALTE6	6.79(7.85)	4.62(7.21)	17	12	1.1604	0.1229
PALTA2	1.83(1.22)	2.31(1.16)	20	9	−1.4759	0.0700
PALTA4	2.93(1.22)	3.10(1.39)	17	12	−0.5743	0.2829
PALTA6	2.07(2.03)	1.28(1.86)	12	17	0.3058	0.0730
PALTA8	0.41(1.23)	0.14(0.74)	4	25		0.3125
PALTA12	0.14(0.74)	0.00(0)	1	28		0.5000

Table S2. Measures obtained from the Motor Screening Task (MOT) and Pattern Recognition Memory (PRM) test, presented as Mean ± SD of the Raw Score.

Type	Pre-treatment	Post-treatment	Participants	Test statistics		
	Mean(SD)	*Mean(SD)*	*N*	*Ties*	*Z*	*P*
MOTML	1119.64(517.89)	1015.88(471.10)	29	0	1.600	0.0548
MOTSDL	376.48(420.68)	282.91(346.31)	29	0	0.5348	0.2963
PRMPCI	61.54(14.90)	64.18(13.24)	27	1	−0.8742	0.1910
PRMMCLI	4828.85(2994.38)	3859.55(2426.25)	26	0	0.7873	0.2155
PRMMDCLI	3739.90(2492.51)	3279.90(2684.65)	26	0	0.5842	0.2796
PRMCLSDI	4570 (8977.97)	2513.36(2019.01)	26	0	0.4318	0.3329

Table S3. Measures obtained from the Reaction Time (RTI) test, presented as Mean ± SD of the Raw Score.

Type	Pre-treatment	Post-treatment	Participants	Test statistics		
	Mean(SD)	*Mean(SD)*	*N*	*Ties*	*Z*	*P*
RTISMRT	471.22(132.66)	526.58(187.65)	28	0	−0.8767	0.1903
RTISMDRT	429.80(96.19)	458.79(144.16)	27	1	−0.3244	0.3728
RTISRTSD	137.35(144.25)	213.37(224.78)	28	0	−1.3695	0.0854
RTISMMT	340.25(132.58)	369.47(148.22)	28	0	−0.8881	0.1872
RTISMDMT	323.52(133.32)	351.55(147.15)	28	0	−0.7401	0.2296
RTISMTSD	89.48(152.67)	100.84(102.47)	28	0	−1.7192	0.0428
RTIFMRT	530.25(165.21)	499.03(109.87)	28	0	−0.1252	0.5498
RTIFMDRT	484.93(98.09)	480.59(96.38)	29	0	0.2733	0.3922
RTIFRTSD	149.72(234.69)	104.07(69.43)	28	0	0.8116	0.2084
RTIFMMT	377.74(159.46)	388.58(137.90)	29	0	−0.1936	0.4233
RTIFMDMT	365.24(171.22)	381.95(135.11)	29	0	−0.2162	0.4144
RTIFMTSD	73.79(80.04)	72.57(55.26)	28	0	−1.0133	0.1555
RTISES	6.10(5.63)	6.93(5.06)	28	0	−1.0621	0.1441
RTISESI	2.52(3.00)	2.69(4.05)	21	8	−0.0702	0.4720
RTISESPR	1.72(2.63)	2.66(2.06)	25	4	−2.0286	0.0213
RTISESNR	0.55(0.95)	0.59(1.18)	13	16		0.4054
RTIFES	4.45(4.89)	3.66(5.18)	23	6	0.7173	0.2366
RTIFESI	1.90(1.84)	1.07(1.56)	17	12	1.9365	0.0264
RTIFESNR	0.38(1.05)	0.38(0.73)	7	22		0.4219
RTIFESPR	0.93(1.62)	0.97(2.26)	18	11	0.1113	0.4557

Table S4. Measures obtained from the Spatial Working Memory (SWM) test, presented as Mean ± SD of the Raw Score.

Type	Pre-treatment	Post-treatment	Participants	Test statistics		
	Mean(SD)	*Mean(SD)*	*N*	*Ties*	*Z*	*P*
SWMTE468	25.52(10.24)	23.69(8.06)	28	1	0.6729	0.2505
SWMBE468	24.52(8.78)	22.83(7.52)	27	2	0.7585	0.2241
SWMWE468	3.69(6.69)	2.38(3.70)	23	6	0.8100	0.2090
SWMDE468	2.52(4.709	1.42(2.81)	19	10	1.2057	0.1140
SWMBE4	2.34(1.72)	1.90(1.47)	22	7	1.0032	0.1579
SWMWE4	0.24(0.83)	0.24(0.51)	7	22		0.6094
SWMTE6	6.93(4.15)	7.76(4.64)	26	3	1.4300	0.9236
SWMBE6	6.62(3.55)	7.52(4.32)	26	3	-1.3391	0.0903
SWMDE6	0.83(1.89)	0.83(2.17)	12	17		0.4363
SWMTE4	2.41(1.86)	2.07(1.65)	23	6	0.6480	0.2585
SWMTE8	16.17(5.98)	13.86(3.93)	26	3	1.4756	0.0700
SWMBE8	16.04(4.55)	13.57(3.76)	26	3	1.6287	0.0517
SWMWE8	2.31(3.92)	1.07(1.60)	22	7	1.4228	0.0774
SWMDE8	1.69(2.85)	0.62(1.15)	18	11	1.8728	**0.0305**
SWMTE12	46.97(13.25)	46.71(13.64)	29	0	0.2963	0.3835
SWMBE12	44.82(11.30)	43.89(11.42)	26	3	0.5593	0.2880
SWMWE12	10.57(10.73)	10.04(9.20)	23	6	0.1369	0.5545
SWMS6	4.03(0.73)	4.10(0.86)	17	12	-0.3803	0.3519
SWMS	9.55(1.33)	9.52(1.27)	20	9	-0.0965	0.5385
SWMSX	17.29(2.11)	17.64(2.04)	25	4	-0.6326	0.2635

Table S5. Measures obtained from the Delayed Matching to Sample (DMS) test, presented as Mean ± SD of the Raw Score.

Type	Pre-treatment	Post-treatment	Participants	Test statistics		
	Mean(SD)	*Mean(SD)*	*N*	*Ties*	*Z*	*P*
DMSTC	11.46(4.00)	11.86(3.47)	26	3	−0.4612	0.3223
DMSPCAD	48.57(20.31)	50.54(20.71)	27	2	−0.5350	0.2963
DMSPCS	76.43(32.23)	80.00(21.77)	19	10	−0.2473	0.4023
DMSPC0	61.43(27.18)	63.57(26.14)	18	11	−0.2105	0.4166
DMSPC4	49.29(23.40)	47.14(25.07)	19	10	0.3493	0.3634
DMSPC12	42.14(24.55)	46.43(23.76)	20	9	−0.8708	0.1919
DMSTE	9.82(10.46)	8.17(3.41)	26	3	0.1898	0.4247
DMSTEC	2.68(2.04)	2.39(2.08)	24	5	0.6099	0.2709
DMSTEP	3.82(2.31)	4.07(1.80)	24	5	−0.4780	0.3163
DMSTEAD	6.82(2.89)	7.17(2.82)	26	3	−0.1662	0.4340
DMSTECAD	2.43(1.71)	2.18(1.74)	22	7	0.7001	0.2419
DMSTEPAD	3.32(2.11)	3.36(1.54)	22	7	−0.0327	0.5131
DMSTEDAD	1.07(1.02)	1.61(1.59)	24	5	−1.2833	0.0997
DMSML	4807.77(2558.14)	5415.50(1901.22)	29	0	−1.2325	0.1089
DMSMDL	4161.64(2,045.21)	4689.95(1,646.82)	29	0	−1.1272	0.1298
DMSLSD	2708.73(1855.46)	2601.85(2138.46)	29	0	0.8289	0.2036
DMSMLAD	5060.14(2,322.13)	5573.61(2,562.58)	29	0	−0.4204	0.3371
DMSMDLAD	4029.54(1847.66)	5012.40(2326.16)	29	0	−1.3334	0.0912
DMSMLS	5234.83(3025.82)	4904.06(1665.76)	29	0	−0.2286	0.5904
DMSMDLS	5028.47(3006.56)	4686.94(1874.77)	29	0	−0.0762	0.5304
DMSLSSD	2189.48(1970.48)	1939.31(1323.16)	29	0	0.3286	0.3712
DMSML0	3779.98(1180.21)	4937.99(2407.30)	29	0	−1.6217	0.0524
DMSL0SD	1786.08(1280.64)	2159.71(2214.57)	29	0	−0.2597	0.3975
DMSML4	5449.22(3295.56)	5348.22(1999.81)	29	0	−0.4843	0.3141
DMSMDL4	5338.40(3366.93)	5099.68(2040.76)	29	0	−0.7534	0.2256
DMSL4SD	2834.80(3147.16)	1991.29(1431.78)	29	0	0.8710	0.1919
DMSML12	6730.57(3673.68)	6598.63(4382.03)	29	0	0.8429	0.1997
DMSL12SD	3135.32(3012.96)	2838.02(5176.16)	29	0	1.0083	0.1567
DMSCC	1.61(0.47)	1.69(0.39)	27	2	−0.8269	0.2041
DMSPEGE	0.38(0.19)	0.36(0.22)	25	4	0.2818	0.3891
DMSPEGC	0.45(0.22)	0.43(0.22)	26	3	0.1217	0.4516
